# Highlight: A Deep Dive into the Adaptive Radiation of Clownfish

**DOI:** 10.1093/gbe/evad127

**Published:** 2023-07-15

**Authors:** Casey McGrath

Clownfish, renowned for their vibrant colors and unusual symbiotic relationship with sea anemones, have long captivated the imagination of scientists and nature enthusiasts alike. They also serve as a promising model organism for studying adaptive radiations, as their interactions with sea anemones appear to have triggered their rapid diversification into 28 species. Despite the clownfish's popularity, however, the genetic basis and evolutionary mechanisms behind their extraordinary radiation have remained largely unexplored until now. A new study published in https://doi.org/10.1093/gbe/evad088, provides new insights into the genomic architecture and evolutionary mechanisms that have allowed clownfish to diversify and thrive in various ecological niches (Marcionetti and Salamin 2023).

The study, conducted by Anna Marcionetti and Nicolas Salamin from the University of Lausanne, compared the genome sequences of ten clownfish species grouped into five pairs based on phylogenetic relatedness. Each pair included one generalist clownfish species, which may associate with several different sea anemone hosts, and one specialist species, which inhabits just a single species of anemone. Thus, there was ecological and phenotypic divergence within pairs in terms of host usage, as well as patterns of ecological and phenotypic convergence across pairs. This unique design allowed the researchers to investigate the roles of parallel and convergent evolution following the clownfish radiation.

“Adaptive radiations have always interested me because they can help us understand the mechanisms behind the origin of species,” stated Salamin. “Being able to combine new genomic resources to study in detail the genetic mechanisms of the clownfish radiation is exciting because it can help us understand how this iconic group has evolved and how species have adapted to sea anemones, which is such an intriguing mutualistic interaction.”

The study's findings indicate that hybridization between clownfish lineages has played a significant role in their evolutionary trajectories. Moreover, the study revealed a genome-wide acceleration in evolution among clownfish, with over 5% of all genes found to be under positive selection. This includes several genes that may be linked to the size-based hierarchical social structure unique to clownfish. In clownfish social groups, the breeding female and male are the largest and second-largest individuals, respectively, with nonbreeders becoming gradually smaller as the hierarchy is descended. Genes under positive selection in clownfish included somatostatin, which may control growth related to this size-based social structure; the gene *NPFFR2*, which may influence growth by regulating food intake and appetite; and the receptor for isotocin, which modulates social behavior.

**Fig. 1.**
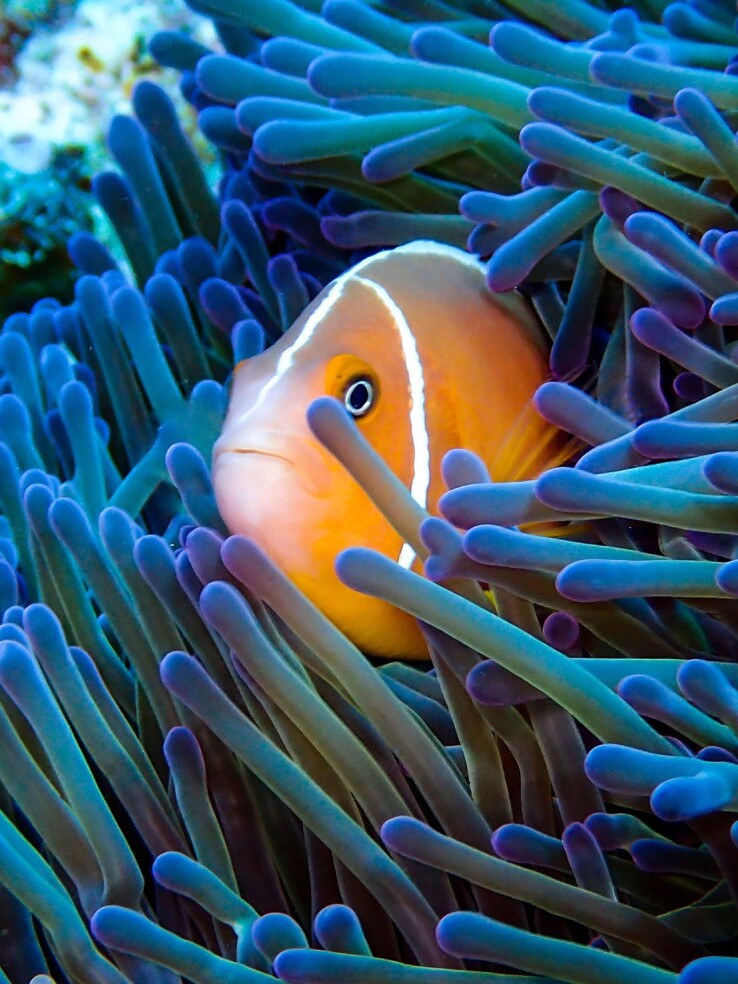
The clownfish *Amphiprion perideraion* in the sea anemone *Heteractis magnifica*. Photograph taken by Sara Heim in New Caledonia.

Positively selected genes also included those involved in adaptation to different ecological niches, such as rhodopsin, a gene that allows for fine-tuning of the visual system at different depths, and the *duox* gene, which regulates the formation of the white stripes that give clownfish their distinct appearance (fig. 1). These findings suggest that the accelerated evolutionary rates observed in clownfish may be associated with the emergence of their unique social and ecological adaptations.

Intriguingly, the study also revealed that generalist clownfish species, which may associate with up to ten different anemone hosts, exhibit faster evolutionary rates than specialist species, which inhabit just a single species of anemone. This may reflect the more diverse or dynamic environments to which the generalists must adapt. Furthermore, the researchers detected genes with parallel patterns of relaxation or intensification of purifying selection in specialist or generalist species, indicating parallel evolution of generalists and specialists to similar ecological niches.

While these results are intriguing, the authors acknowledge the challenges of linking these findings to clownfish phenotypes and the need for future research to fully characterize clownfish ecology and functional traits. “To obtain a full understanding of the radiation of clownfish, it will be essential to achieve a comprehensive characterization of their ecology and functional traits. Nevertheless, this study suggests candidate genes and pathways that may be involved in diversification of the group, providing valuable hints for future functional research.”

In addition, the results of this study can be used to inform future marine conservation and management efforts as they relate to clownfish populations. Understanding the genetic adaptations of clownfish to their environment, including their social structures and interactions with sea anemones, can aid in the development of targeted conservation interventions to mitigate the impacts of environmental stressors and promote the long-term survival of clownfish populations. This study highlights the importance of considering the genetic aspects of a species’ biology when formulating conservation plans and underscores the need for continued research and conservation efforts to safeguard these iconic marine species.
